# Investigating the Relationship Between Resilience, Stress-Coping Strategies, and Learning Approaches to Predict Academic Performance in Undergraduate Medical Students: Protocol for a Proof-of-Concept Study

**DOI:** 10.2196/14677

**Published:** 2019-09-19

**Authors:** Yajnavalka Banerjee, Aya Akhras, Amar Hassan Khamis, Alawi Alsheikh-Ali, David Davis

**Affiliations:** 1 College of Medicine Mohammed Bin Rashid University of Medicine and Health Sciences Dubai United Arab Emirates; 2 Center for Outcomes and Research in Education Mohammed Bin Rashid University of Medicine and Health Sciences Dubai United Arab Emirates; 3 Center for Medical Education University of Dundee Nethergate, Dundee United Kingdom; 4 Hamdan Bin Mohammed College of Dental Medicine Mohammed Bin Rashid University of Medicine and Health Sciences Dubai United Arab Emirates; 5 Department of Family and Community Medicine University of Toronto Toronto, ON Canada

**Keywords:** medical education, undergraduate medical education, psychological resilience, resilience, learning, coping behavior, psychological stress

## Abstract

**Background:**

The evolution of an undergraduate medical student into an adept physician is perpetual, demanding, and stressful. Several studies have indicated medical students have a higher predominance of mental health problems than other student groups of the same age, where medical education acts as a stressor and may lead to unfavorable consequences such as depression, burnout, somatic complaints, decrease in empathy, dismal thoughts about quitting medical school, self harm and suicidal ideation, and poor academic performance. It is imperative to determine the association between important psychoeducational variables and academic performance in the context of medical education to comprehend the response to academic stress.

**Objective:**

The aim of this proof-of-concept study is to determine the relationship between resilience, learning approaches, and stress-coping strategies and how they can collectively predict achievement in undergraduate medical students. The following research questions will be addressed: What is the correlation between the psychoeducational variables resilience, learning approaches, and stress-coping strategies? Can academic performance of undergraduate medical students be predicted through the construction of linear relationships between defined variables employing the principles of empirical modeling?

**Methods:**

Study population will consist of 234 students registered for the MBBS (Bachelor of Medicine, Bachelor of Surgery) at Mohammed Bin Rashid University of Medicine and Health Sciences distributed over 4 cohorts. Newly registered MBBS students will be excluded from the study. Various psychoeducational variables will be assessed using prevalidated questionnaires. For learning approaches assessment, the Approaches and Study Skills Inventory for Students questionnaire will be employed. Resilience and stress-coping strategies will be evaluated using the Wagnild-Young resilience scale and a coping strategies scale derived from Holahan and Moos’s Coping Strategies Scale, respectively. Independent variables (resilience, stress-coping strategies, and learning approaches) will be calculated. Scores will be tested for normality by using the Shapiro-Wilk test. An interitem correlational matrix of the dependent and independent variables to test pairwise correlation will be formed using Pearson bivariate correlation coefficients. Regression models will be used to answer our questions with type II analyses of variance in tests involving multiple predictors. Regression analyses will be checked for homogeneity of variance (Levine test) and normality of residuals and multicollinearity (variance inflation factor). Statistical significance will be set at 5% (alpha=.05). Effect sizes will be estimated with 95% CIs.

**Results:**

Psychoeducational instruments in the form of validated questionnaire have been identified in relation to the objectives. These questionnaires have been formatted for integration into Google forms such that they can be electronically distributed to the consenting participants. We submitted the proposal to MBRU institutional review board (IRB) for which exemption has been awarded (application ID: MBRU-IRB-2019-013). There is no funding in place for this study and no anticipated start date. Total duration of the proposed research is 12 months.

**Conclusions:**

Psychoeducational instruments used in this study will correlate resilience, stress-coping strategies, and learning approaches to academic performance of undergradudate medical students. To the best of our knowledge, no study exploring the multidimensional association of key psychoeducational variables and academic performance in undergraduate medical students has been pursued. Investigated variables, resilience, learning approaches, and stress-coping strategies, are individual traits, however; students’ learning history before they joined MBRU is unknown, so our research will not be able to address this specific aspect.

**International Registered Report Identifier (IRRID):**

PRR1-10.2196/14677

## Introduction

### Background

The evolution of an undergraduate medical student into a safe and competent physician is interminable, demanding, and stressful [1]. This journey involves combatting what Smith [2] defined as the “swampy lowlands where situations are confusing ‘messes’ incapable of technical solutions” coping with hidden insecurities of clinical practice. In fact, medical students have a higher predominance of mental health problems than other student groups of the same age [3]. This indicates that medical education itself contributes as a key stressor, an observation corroborated by other studies [4,5]. Academic stress is defined as the body’s response to academic-related strains and tensions that exceed adaptive potentials of students. Medical students experience high degrees of academic stress, where the most commonly reported stressors in the academic environment are related to oral presentations, academic overload, scarcity of time to meet commitments, and taking examinations. [6]. While some academic stress may boost academic performance [7], elevated stress levels in medical students may lead to detrimental consequences such as depression [8], burnout [9], somatic complaints [10], decrease in empathy [11], dismal thoughts about quitting medical school [12], suicidal ideation [5], and poor academic performance [13,14]. Therefore, it is imperative to determine the association between important psychoeducational variables and academic performance in the context of medical education to define the role of each psychoeducational variable in the response to academic stress.

### Aim

The aim of this proof-of-concept study is to determine the relationship between meta-motivational skills for handling stress (resilience), meta-cognitive skills for study (learning approaches), and meta-emotional skills for managing stress (stress-coping strategies) and how they can collectively predict achievement in undergraduate medical students, founded on the competence of learning, studying, and performing under stress (CLSPS) model [15] (Textbox 1).

### Research Questions

The following research questions will be addressed:

- What is the correlation between the psychoeducational variables resilience, learning approaches, and stress-coping strategies in an undergraduate entry medical program?
- Can academic performance of undergraduate medical students be predicted through the construction of linear relationships between the defined variables employing the principles of empirical modeling?

### Hypotheses

The research questions are founded on the following hypotheses regarding undergraduate medical students:

- Resilience is correlated positively with strategic and deep learning approaches and negatively with surface learning approaches.
- Resilience is correlated positively with problem-focused stress-coping strategies and negatively with emotion-focused stress-coping strategies.
- Emotion-focused stress-coping strategies are correlated positively with surface learning approaches and negatively with strategic and deep learning approaches.
- Resilience is a positive predictor of strategic and deep learning approaches and a negative predictor of surface learning approaches; in addition, it is a positive predictor of problem-focused stress-coping strategies and a negative predictor of emotion-focused stress-coping strategies. Moreover, resilience together with strategic and deep learning approaches and problem-focused stress-coping strategies will have a positive and a linear relationship with academic performance.

**The competence of learning, studying, and performing under stress model of de la Fuente [15].**

Knows (knowledge):

- Facts: knowledge about the characteristics of the class subject or professional exam (career opportunities, percentage of candidates who pass, requirements)
- Concepts: competitive exam system, requirements, type of examination, scoring, prior merits/credits, type of class subject
- Principles: beliefs about the professional exam or selection process

Knows how (skills):

- Principles: beliefs about the professional exam or selection process
- Instrumental skills: written and oral skills
- Learning and study skills: study skills and techniques
- Meta-cognitive skills for study: learning approaches
- Meta-emotional skills for managing stress: coping strategies
- Meta-motivational skills for managing stress: resilience
- Meta-behavioral skills for managing stress: self-regulation strategies

Knows how to be (attitudes):

- Attitudes and values: behavioral confidence, achievement motivation, mindset
- Study habits (time management, persistence, discipline)

### Literature Review

#### Search Strategy

Relevant publications were searched in PubMed using the keywords resilience, learning approaches, stress-coping strategies, academic performance, and combinations and variations of these words in conjunction with PubMed-accepted Boolean operators employing the strategy of Jadad et al [16]. Further, this proposal has drawn from the references listed in the research of de la Fuente et al [17] and Garzon-Umerenkova et al [18].

#### Meta-Motivational Variable: Resilience

Resilience is “a dynamic process wherein individuals display positive adaptation despite experiences of significant adversity or trauma” [19]. In medical education, resilience has a key role as a motivational-affective variable, where it not only acts as a key impetus for the comprehension of scholastic and individual objectives, it also provides one with suitable strategies to tackle adverse conditions of stress and anxiety [20]. Erudition directed to superior academic performance in medical students not only requires motivation, effectively tackling rhythms, modular stresses, and responses of different types but also the capability to self-motivate to effectively counter taxing and traumatic situations, concurrently avoiding circumstances of exacerbation or poignant distress such as vulnerability, apathy, dejection, or anguish [21-23]. Although considerable research has focused on investigating the factors for resilience in medical education, little research has been conducted to investigate the relationship of resilience with other confounding psychoeducational elements such as stress-coping strategies and learning approaches.

#### Meta-Emotional Variable: Stress-Coping Strategies

Folkman and Moscowitz [24] define coping as “continually changing cognitive and behavioral practices that are developed to handle specific external and/or internal demands that are valued as beyond the individual’s resources.” Individuals cope with different traumatic and taxing circumstances in a manner that surpasses the effect of the situational and chronological context, a phenomenon often referred to as coping styles [25]. Intrinsic to these styles is the involvement of a defined thought process and actions defined by Soucy as stress-coping strategies [26]. These can be broadly classified into three types of strategies [27]: problem-focused, emotion-focused, and avoidance-focused.

Although considerable research has been pursued to characterize the coping strategies of medical students, including a 10-year longitudinal study to predict how coping strategies inform styles of success in medical careers [28], there is a dearth of studies investigating how stress-coping strategies can predict academic performance in a multidimensional milieu (in association with resilience and learning approaches).

#### Meta-Cognitive Variable: Learning Approaches

**Figure 1:**
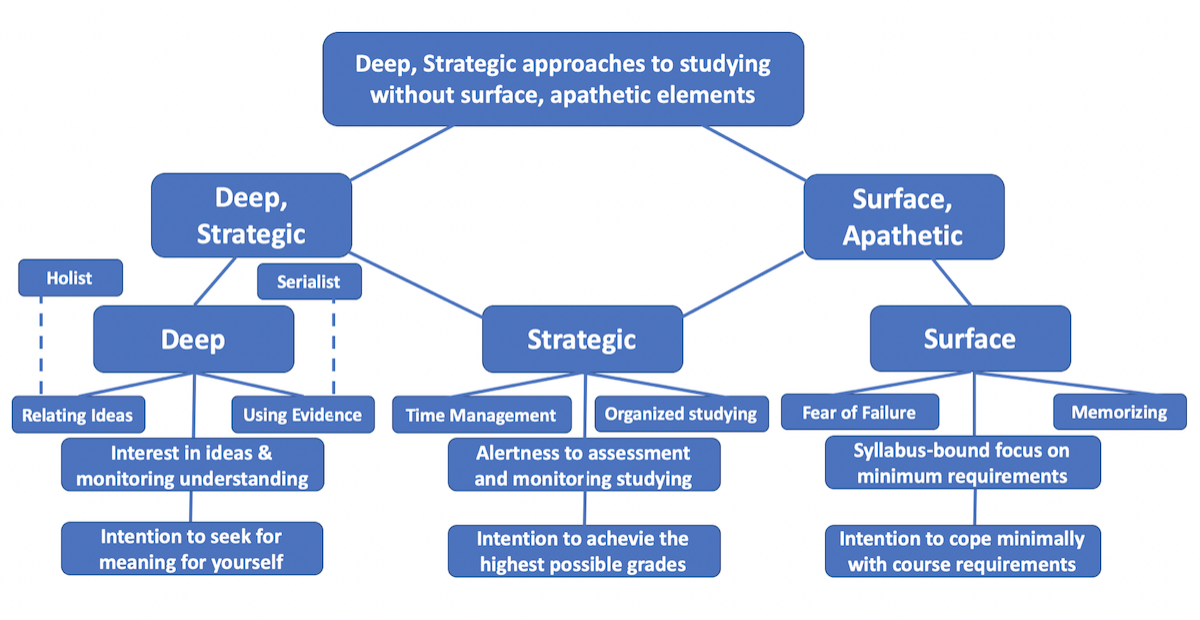
Classification of learning approaches as defined by Entwistle [30].

Biggs defines learning approaches as “learning processes that emerge from students’ perceptions of academic tasks influenced by their personal characteristics” [29]. Entwistle et al [30] describe 3 learning approaches: deep, surface, and strategic [30] (Figure 1). The deep approach characterizes students who intend to seek meaning for themselves as well as relating ideas and using evidence. The strategic approach involves students who intend to excel academically by organized studying in order to achieve the highest possible grades. The surface apathetic approach has the intention of coping with the minimum course requirements and is linked to rote memorizing and a fear of failure [31].

### Preliminary Data From Initial Study

An initial cross-sectional study was pursued at MBRU. The ASSIST questionnaire [30] was circulated to 84 students in the college of medicine. Of the 84 students, 64 responded to the questionnaire. Of the 64 responses, 4 responses were excluded as they were either incomplete or ambiguous. Statistical analysis was conducted on the data obtained from 60 responses. Of these students, 57% (34/60) used a deep learning approach, 16% (10/60) used a strategic learning approach, and 27% (16/60) used a surface learning approach.

**Figure 2:**
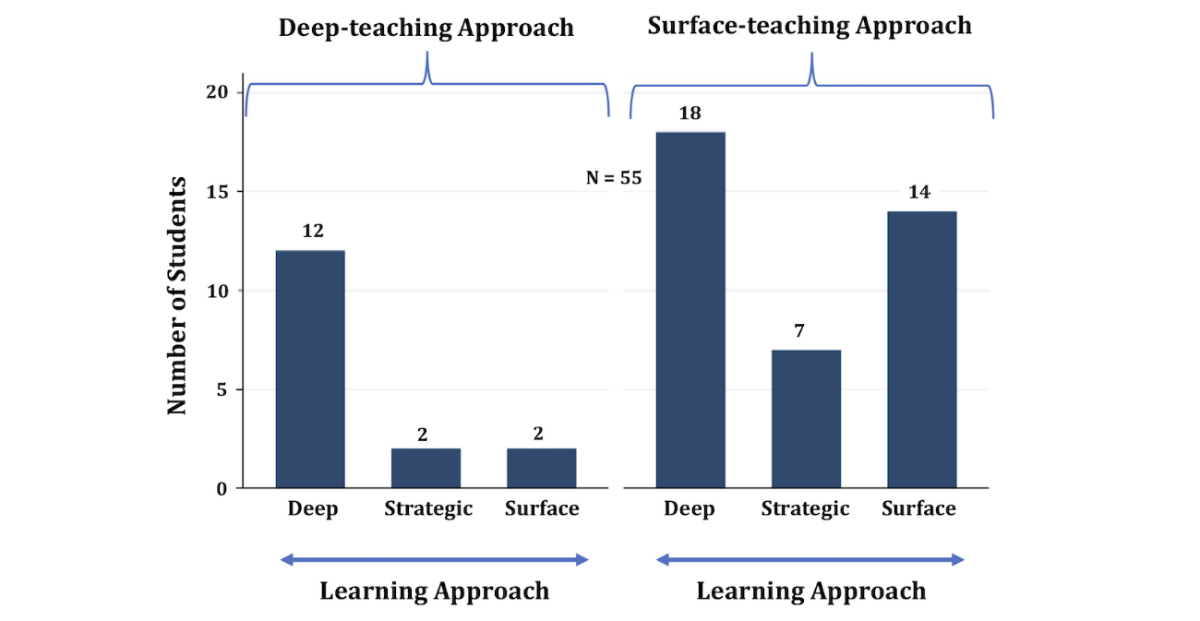
Preliminary data showing the frequency of predominant learning approaches classified by predominant teaching approaches.

Next, we investigated the association of learning approaches with teaching approaches in the 60 responses using logistic regression. In this analysis, responses of 5 students were excluded, as they had equivalent scores in teaching approaches. Among the 55 included students, 71% (39/55) preferred the surface teaching approach and 29% (16/35) preferred the deep teaching approach. Furthermore, strategic learners had a significant positive correlation with perceived academic performance compared with other learners (Figure 2).

This initial study indicated that while the predominant learning approach was deep learning (seeking meaning and critical thinking), the preferred teaching approach was surface teaching. Also, strategic learners perceive themselves to perform better academically (Tables 1 and 2) [32].

**Table 1:** Correlation of average learning approach scores and academic performance.

Correlation variable	Strategic score	Surface score	Deep score	Academic performance
Strategic score	—^a^	—	—	—
Surface score	–.08	—	—	—
Deep score	.38	–.32	—	—
Academic performance	.54	–.25	.23	—

^a^Not applicable.

**Table 2:** *P* values^a^ of correlation of average learning approach scores and academic performance.

Correlation variable	Strategic score	Surface score	Deep score	Academic performance
Strategic score	>.99	—^b^	—	—
Surface score	.56	>.99	—	—
Deep score	.003	.01	>.99	—
Academic performance	<.001	.06	.08	>.99

^a^Spearman correlation.

^b^Not applicable.

However, what happens in a multidimensional milieu, when resilience and stress-coping strategies of students are also included, is currently unknown and will be investigated in this study.

### Rationale for Proposed Research

In the demanding academic milieu of medical education, the psychoeducational variables resilience, learning approaches, and stress-coping strategies act in concert; it is imperative to investigate the relationships between these variables and their collective effect on the academic performance of undergraduate medical students. To our knowledge this has not been studied, and the proposed research would address this gap.

## Methods

### Study Landscape

**Figure 3:**
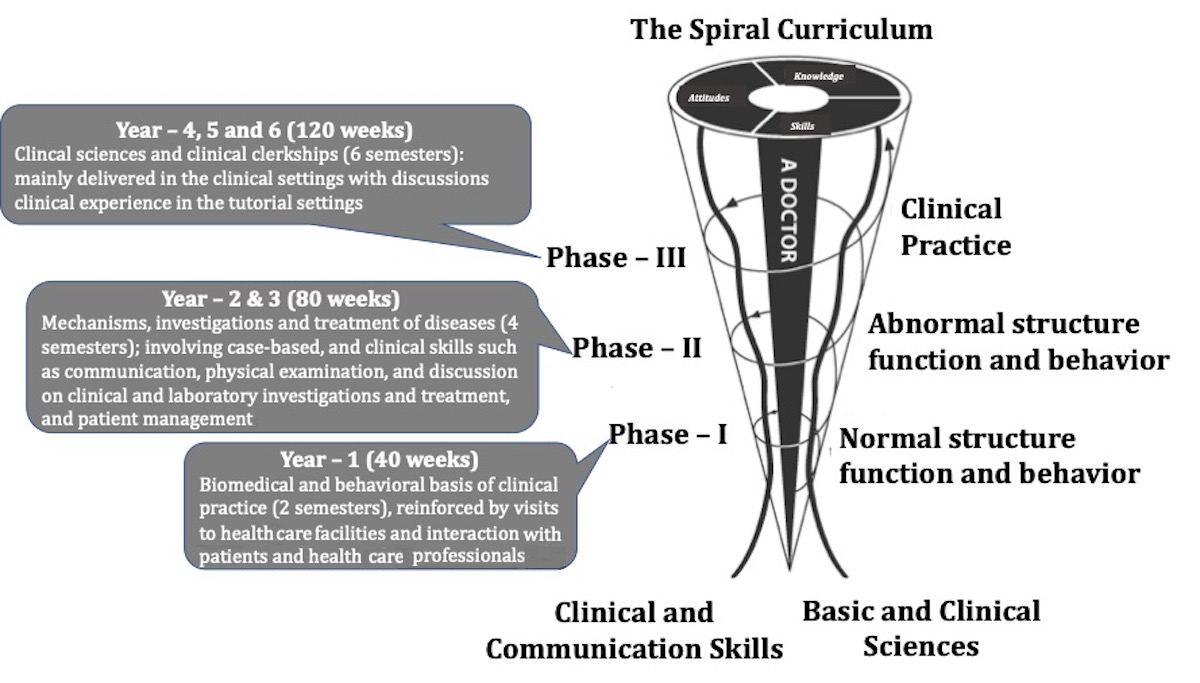
The 6-year undergraduate medical curriculum at Mohammed Bin Rashid University of Medicine and Health Sciences is divided into three phases. Each phase of the curriculum includes integrated courses and builds on the preceding one such that the curriculum is a spiral: the students repeat the study of a subject, each time at a higher level of difficulty and in greater depth.

Mohammed Bin Rashid University of Medicine and Health Sciences (MBRU) is a new medical school located in Dubai Health Care City, the health care hub of United Arab Emirates, with a 6-year MBBS (Bachelor of Medicine, Bachelor of Surgery, or *Medicinae Baccalaureus Baccalaureus Chirurgiae*) undergraduate entry medical program where the curriculum is founded on a competency-based educational model. The MBRU curriculum is divided into 3 phases (Figure 3). Each phase of the curriculum includes integrated courses and builds on the preceding one such that the curriculum is a spiral: the students repeat the study of a subject, each time at a higher level of difficulty and in greater depth. The school has a diverse student population, drawing students from more than 19 countries across the globe. Approximately 75% of the students are women.

### Participants

As indicated earlier, MBRU is a new medical school in its fourth year of operation. The study population will consist of 234 MBBS students (students in the dental program are not eligible) distributed over four cohorts. Purposive sampling will be used. Newly registered MBBS students will be excluded from the study because their brief period in the program is not adequate to evaluate their academic performance and psychoeducational variables.

Mapping of the correlation coefficient values (over a defined range from .20 to .80) for association of resilience to academic performance with a range of power values (0.60 to 0.90; Table 3) indicates that the number of participants will be suitable for the statistical correlations performed in the study. To detect a simple correlation between resilence and academic performance, where correlation coefficient *r*=.20 of N observations using a 2-sided test of 5% significance level (alpha=.05) with 80% power (β=0.20), the required sample size is approximately 194.

### Learning Approaches Evaluation

Our study will employ a modification of the Approaches and Study Skills Inventory for Students (ASSIST; Multimedia Appendix 1) questionnaire [33] to evaluate the predominant learning approaches of the students. This questionnaire was developed by Entwistle et al [30] to evaluate approaches to learning and has been refined and improved based on educational philosophies put forth by Martin and Saljo [34] and others [29].

The initially published ASSIST questionnaire consists of sections A, B, and C with questions rated on a Likert scale. As shown in Table 4, in the proposed research the 66 items have been reduced to 41 items (sections B and C). We modified the questionnaire to decrease the number of questions while maintaining an equal number of questions across the learning approaches. In the modified ASSIST, 4 items have been added to record the demographics of participants (age, gender, year of study in the MBBS program, and high school education). In modifying the ASSIST questionnaire, care has been taken such that overall validity of the tool is preserved. The original ASSIST questionnaire [30] has been amended instead of using an existing abridged version to make the tool as relevant as possible to the context of the proposed research. Modifications have been introduced to avoid survey fatigue among participants; specific items not relevant to the participants have been removed. Additionally, upon piloting the questionnaire we found that the language in a few items was ambiguous, and we amended these items.

**Table 3:** Power calculation table.

Power of the study (1-β)	Sample correlation (*r*)						
	.20	.30	.40	.50	.60	.70	.80
0.90	259	113	62	38	25	17	12
0.80	194	85	47	29	19	13	10
0.70	153	67	37	23	16	11	8
0.60	122	54	30	19	13	10	7

**Table 4:** Modified Approaches and Study Skills Inventory for Students questionnaire to be implemented in this study.

Section	Content	Scale	Modifications
A	Conceptions of learning	Likert scale (1-5)	Removed a-f
B	Approaches to studying	Likert scale (1-5)	33/52 questions included (11 per learning approach)
C	Preferences for different types of courses and teaching	Likert scale (1-5)^a^	No modifications (9 total questions)
Added	Demographics	Continuous	Age, gender, year of study, high school education

^a^Last question: perceived academic performance rating (1-9).

### Resilience Evaluation

The Connor-Davison [35] and Wagnild-Young [36] resilience scales were considered for our evaluation. We chose the Wagnild-Young scale (Multimedia Appendix 2) because it was developed using an oblimin rotation factor analysis (allowing correlatation) where the factor construction characterizes personal competence and acceptance of self and life [37]. The scale consists of 14 Likert-scale items grouped in five domains: self-reliance, meaning, equanimity, perseverance, and existential aloneness.

### Coping Strategies Evaluation

A 13-item coping strategies questionnaire has been designed (Multimedia Appendix 3) that will assess participant cognitive, emotional, and behavioral approaches for tackling difficulties and problems. The cognitive and emotional approaches (items 2, 3, and 4) have been adapted from the Coping Strategies Scale of Holahan and Moos [38]. Additional items focusing on emotional and cognitive approaches (items 1, 5, 6, and 8) have been adapted from Hamby et al [39]. Other items in the questionnaire are from Spitzberg et al [40].

### Data Collection Procedure

Students participating in the proposed research will receive identical information, disseminated through Google Forms, similar to previous research studies conducted in medical education at MBRU [41-43]. Participants will respond to the questionnaires during the self-study time between 11 am and 1 pm (after the morning teaching sessions). Gathering and processing of the collected data will be pursued with the informed consent of the participating students, in line with the ethical and deontological principles of psychology. Collected data will be analyzed in an anonymous and group format, and the data will be stored in an encrypted database and on a password-protected solid-state drive with the research team.

We submitted the proposal to MBRU institutional review board (IRB) for which exemption has been awarded (application ID: MBRU-IRB-2019-013). Further clarification with regard to the policies and terms of reference can be obtained from the IRB.

### Data Analysis

The questionnaire response files from Google Forms will be converted to a spreadsheet, and the questions for each approach will be organized into adjacent columns with the value of each response in the respective row. All collected data will be cross-verified by two investigators from the research team. The average score for each questionnaire will be generated for each student. This will be done by taking the average of all responses recorded for a certain questionnaire for a particular student.

SPSS Statistics for Windows version 23.0 (IBM Corp) will be used for all statistical analyses. Cronbach alpha will be used to check internal consistency, and explanatory factor analysis will be used specifically for the questionnaire to confirm the evidence of its validity in the literature. Outliers will be identified by using a Bonferroni outlier test (*P*<.05). Scores of independent variables (resilience, stress-coping strategies, and learning approaches) will be calculated for independent variables. All scores will be tested for normality by using the Shapiro-Wilk test. An inter-item correlational matrix of the dependent and independent variables to test the pairwise correlation will be formed using Pearson bivariate correlation coefficients. Regression models will be used to answer questions with type II analyses of variance in tests involving multiple predictors. Regression analyses will be checked for homogeneity of variance (Levine test), normality of residuals, and multicollinearity (variance inflation factor). Statistical significance will be set at the conventional 5% threshold (alpha=.05). Effect sizes will be estimated with 95% CIs.

Ordered logistic regression will be used to analyze the effect of individual psychoacademic variables on academic performance controlling for age, gender, and cohort to which the student belongs.

### Ethical Considerations

#### Distributive Justice

Distributive justice in medical education in line with the concept of egalitarianism [44] dictates that all subjects in the study population are provided with just and equal opportunity to participate in the study [45]. The principal investigator is the course director/instructor for several courses across different student cohorts and therefore has regular and extensive interactions with students. Due to this interaction with students, he may develop the preconceived notion that certain students in these cohorts, because of their personality traits, should not participate in the study. As a result, these students may be inadvertently left out if the recruitment of study participants is pursued by the principal investigator. To address this, participation of students from individual cohorts will be overseen by student representatives randomly assigned from each cohort.

#### Beneficience

Although studies pertaining to the “July phenomenon” (rise in the morbidity and mortality of patients with the inflow of new medical trainees) [46,47] have been unfounded, they have raised concerns regarding beneficence in medical education research. Keeping in mind the key aspects of beneficence, this study includes validated questionnaires and analytical tools and methodologies that have been used in other similar studies without any untoward physiological and psychological effect on the participants. Also, recruitment of participants will involve the use of smart applications (relying less on human involvement), which will minimize harm to the participants from relatively inexperienced researchers (students overseeing recruitment, etc).

#### Power Differential

The power differential under the five bases of power (coercive, reward, legitimate, referent, and expert [48]) must be considered because one of the principal investigators is both the primary researcher and the course director/instructor for several courses across different student cohorts in the study. To prevent such a power differential from compelling subjects to participate in the study, recruitment and associated processes will be carried out by a faculty member from the MBRU school of dentistry, who isn’t in a power relationship with the undergraduate medical students.

#### Respect for Participants

One of the key aspects of the Helsinki declaration on ethical guidance on research involving human subjects requires researchers to acknowledge autonomy of study participants and protect those with diminished autonomy [49]. Therefore, data will be obtained only from consenting participants. All study participants will be required to sign a consent form.

#### Confidentiality

Each participant will be assigned a unique study identifier. No participant names will be collected. Participant responses will be de-identified or anonymized and reported in aggregate. A repository will be created containing participant responses, which will be encrypted and password-protected.

## Results

This study is at the protocol development stage only, and as such, no results are available. The psychoeducational instruments in the form of validated questionnaires have been identified in relation to the objectives. These questionnaires have been formatted for integration into Google Forms such that they can be electronically distributed to the consenting participants.

**Figure 4:**
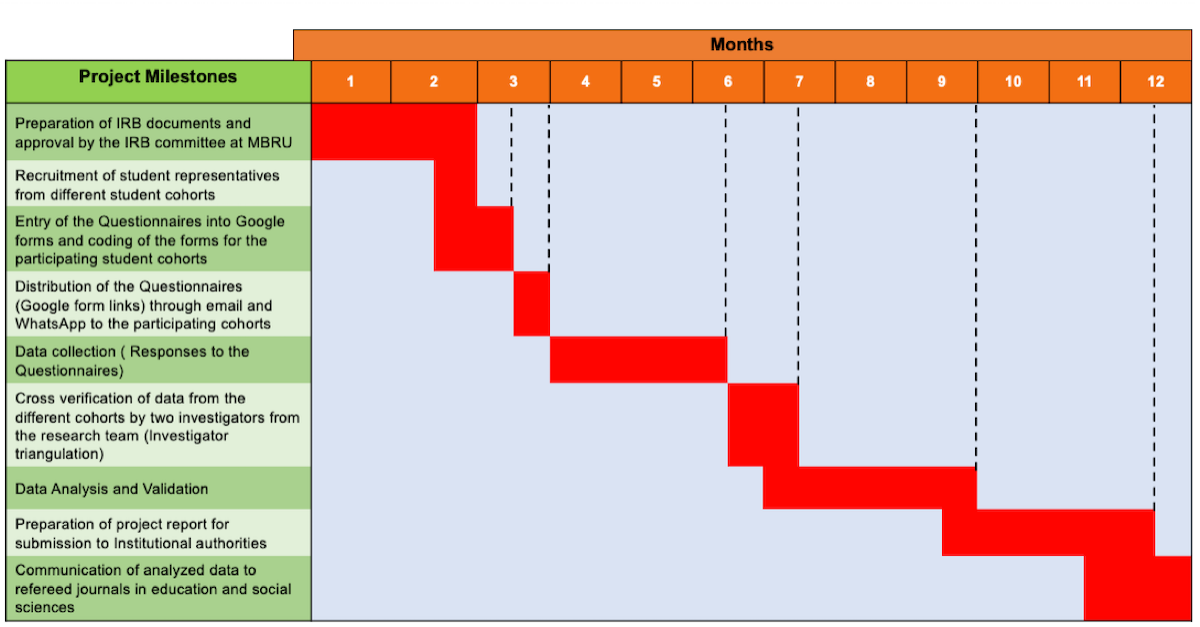
Important project milestones. IRB: institutional review board; MBRU: Mohammed Bin Rashid University of Medicine and Health Sciences.

The MBRU IRB reviewed this study and provided an exempt status (MBRU-IRB-2019-013). Further clarification and information can be obtained from the MBRU IRB at irb@mbru.ac.ae. There is no funding in place for this study and no anticipated start date. The total duration of the proposed research is 12 months. Key project milestones and timeline are shown in Figure 4.

## Discussion

### Summary

We have presented the data from an initial study, where we have correlated student learning approach to academic performance. Data from this initial study further encouraged us to investigate academic performance of medical students in a multidimensional setting; when resilience and stress-coping strategies are also included, the study protocol for this investigation is presented in this article.

Findings from this investigation will elaborate on the need for further research regarding resilience in medical students and how resilience can be improved in this population and emphasize that the concepts of stress, burnout, resilience, and coping appear to be very much related in the context of undergraduate medical education.

### Limitations

The investigated variables, resilience, learning approaches, and stress-coping strategies, are individual traits; students’ learning history before they joined MBRU is unknown, so our research will not be able to address this specific aspect. Investigating this aspect would be difficult as the MBRU student pool draws from 19 different countries and 15 different high school curricula.

In addition, we cannot consider the gender variable in this study as more than 80% of our students are women, which has shown to have an effect on the investigated variables [50,51].

### Conclusions

Results from the different psychoeducational instruments will institute the associative and extrapolative multidimensionality of the different variables in envisaging academic performance of medical students. To our knowledge, no study exploring the multidimensional association of learning approach, resilience, stress-coping strategies, and academic performance in undergraduate medical students has been pursued [52].

Additionally, this research will validate and expand on previous research on the importance of resilience and its association with academic stress and coping strategies in medical students [53,54]. Study results may initiate a framework for assessing psychoeducational variables while admitting students to medical school or during counseling as part of psychoeducational services.

Future studies from this research should investigate the associations of the studied psychoeducational variables with academic emotions or insufficient approaches of stress management, [55-57]. Study results can initiate strategies to integrate the studied variables in different models of medical curricula [58,59], adding to the understanding of the role of meta-motivational and meta-affective approaches during learning in medical school [60].

## Appendix

Multimedia Appendix 1 Modified Approaches and Study Skills Inventory for Students [32] questionnaire.

Multimedia Appendix 2 The 25-item Resilience Scale of Wagnild and Young [35].

Multimedia Appendix 3 The 13-item Coping Scale.

## Abbreviations

ASSIST: Approaches and Study Skills Inventory for Students
CLSPS: competence of learning, studying, and performing under stress
IRB: institutional review board
MBBS: Bachelor of Medicine, Bachelor of Surgery
MBRU: Mohammed Bin Rashid University of Medicine and Health Sciences
