# Supplementary material for: Investigating the Relationship Between Resilience, Stress-Coping Strategies, and Learning Approaches to Predict Academic Performance in Undergraduate Medical Students: Protocol for a Proof-of-Concept Study
Source: JMIR Res Protoc. 2019 Sep 19;8(9):e14677. doi: 10.2196/14677 (PMC6754686; doi:10.2196/14677)
Supplement: Multimedia Appendix 1 [file resprot_v8i9e14677_app1.pdf]

The modified ASSIST questionnaire which will be used in this study (*Note*: The modifications introduced are listed in Table 4 (refer below); the rationale for the modifications is discussed in text under the heading Evaluation of Learning Approaches)

Item no.	Item	1	2	3	4	5
Approaches to Studying						
1	I organise my study time carefully to make the best use of it.					
2	I find I have to concentrate on just memorising a good deal of what I have to learn					
3	Often I feel I'm drowning in the sheer amount of material we're having to cope with					
4	I look at the evidence carefully and try to reach my own conclusion about what I'm studying					
5	It's important for me to feel that I'm doing as well as I can on my courses*					
6	I try to relate ideas I come across to those in other topics or other courses whenever possible					
7	I tend to read very little beyond what is actually required to pass					

8	Regularly I find myself thinking about ideas from lectures when I'm doing other things					
9	I think I'm quite systematic and organised when it comes to revising for exams					
10	Much of what I'm studying makes little sense: it's like unrelated bits and pieces					
11	When I'm working on a new topic, I try to see in my own mind how all the ideas fit together.					
12	I often worry about whether I'll ever be able to cope with the work properly					
13	Often I find myself questioning things I hear in lectures or read in books					
14	I concentrate on learning just those bits of information I have to know to pass					
15	I find that studying academic topics can be quite exciting at times					
16	I keep in mind who is going to mark an assignment and what they're likely to be looking for.					
17	I work steadily through the term or semester, rather than leave it all until the last minute					

**18**	**I'm not really sure what's important in lectures so I try to get down all I can.**					
**19**	**Ideas in course books or articles often set me off on long chains of thought of my own**					
**20**	**Before starting work on an assignment or exam question, I think first how best to tackle it**					
**21**	**I often seem to panic if I get behind with my work.**					
**22**	**When I read, I examine the details carefully to see how they fit in with what's being said.**					
**23**	**I put a lot of effort into studying because I'm determined to do well.**					
**24**	**I gear my studying closely to just what seems to be required for assignments and exams**					
**25**	**Some of the ideas I come across on the course I find really gripping.**					
**26**	**I usually plan out my week's work in advance, either on paper or in my head**					
**27**	**I keep an eye open for what lecturers seem to think is important and concentrate on that**					

**28**	**I generally make good use of my time during the day**					
**29**	**I often have trouble in making sense of the things I have to remember**					
**30**	**When I finish a piece of work, I check it through to see if it really meets the requirements**					
**31**	**It's important for me to be able to follow the argument, or to see the reason behind things**					
**32**	**I like to be told precisely what to do in essays or other assignments**					
**33**	**I sometimes get 'hooked' on academic topics and feel I would like to keep on studying them.**					
**Preferences for different types of course and teaching**						
**34**	**lecturers who tell us exactly what to put down in our notes**					
**35**	**lecturers who encourage us to think for ourselves and show us how they themselves think**					
**36**	**exams which allow me to show that I've thought about the course material for myself**					
**37**	**exams or tests which need only the material provided in our lecture notes**					

**38**	**courses in which it's made very clear which books we should read or refer to***					
**39**	**courses where we're encouraged to read around the subject a lot for ourselves**					
**40**	**books which challenge you and provide explanations which go beyond the lectures**					
**41**	**books which give you definite facts and information which can easily be learned**					
**Academic Progress**		**1**				**9**
**42**	**Finally, how well do you think you have been doing in your assessed work overall, so far?**<br>**Please rate yourself objectively, based on the grades you have been obtaining**					
