# Supplementary material for: Investigating the Relationship Between Resilience, Stress-Coping Strategies, and Learning Approaches to Predict Academic Performance in Undergraduate Medical Students: Protocol for a Proof-of-Concept Study
Source: JMIR Res Protoc. 2019 Sep 19;8(9):e14677. doi: 10.2196/14677 (PMC6754686; doi:10.2196/14677)
Supplement: Multimedia Appendix 2 [file resprot_v8i9e14677_app2.pdf]

The 25-item resilience scale of Wagnild and Young

Item no.	Item	1	2	3	4	5	6	7
1	When I make plans, I follow through with them							
2	I usually manage one way or another							
3	I am able to depend on myself more than anyone else							
4	Keeping interested in things is important to me							
5	I can be on my own if I have to							
6	I feel proud that I have accomplished things in life							
7	I usually take things in stride							
8	I am friends with myself							
9	I feel that I can handle many things at a time							
10	I am determined							
11	I seldom wonder what the point of it all is							
12	I take things one day at a time							
13	I can get through difficult times because I've experienced difficulty before							

**14**	**I have self-discipline**							
**15**	**I keep interested in things**							
**16**	**I can usually find something to laugh about**							
**17**	**My belief in myself gets me through hard times**							
**18**	**In an emergency, I'm someone people can generally rely on**							
**19**	**I can usually look at a situation in a number of ways**							
**20**	**Sometimes I make myself do things whether I want to or not**							
**21**	**My life has meaning**							
**22**	**I do not dwell on things that I can't do anything about**							
**23**	**When I'm in a difficult situation, I can usually find my way out of it**							
**24**	**I have enough energy to do what I have to do**							
**25**	**It's okay if there are people who don't like me**							
