# Supplementary material for: Investigating the Relationship Between Resilience, Stress-Coping Strategies, and Learning Approaches to Predict Academic Performance in Undergraduate Medical Students: Protocol for a Proof-of-Concept Study
Source: JMIR Res Protoc. 2019 Sep 19;8(9):e14677. doi: 10.2196/14677 (PMC6754686; doi:10.2196/14677)
Supplement: Multimedia Appendix 3 [file resprot_v8i9e14677_app3.pdf]

The 13-item Coping Scale (*4 = Mostly true about me; 3 = Somewhat true about me; 2 = A little true about me; 1 = Not true about me*)

Item No.	Item	4	3	2	1
**1**	**When dealing with a problem, I spend time trying to understand what happened**				
**2**	**When dealing with a problem, I try to see the positive side of the situation.**				
**3**	**When dealing with a problem, I try to step back from the problem and think about it from a different point of view.**				
**4**	**When dealing with a problem, I consider several alternatives for handling the problem.**				
**5**	**When dealing with a problem, I try to see the humor in it.**				
**6**	**When dealing with a problem, I think about what it might say about bigger lifestyle changes I need to make.**				
**7**	**When dealing with a problem, I often wait it out and see if it doesn't take care of itself.**				

**8**	**When dealing with a problem, I often try to remember that the problem is not as serious as it seems.**				
**9**	**When dealing with a problem, I often use exercise, hobbies, or meditation to help me get through a tough time.**				
**10**	**When dealing with a problem, I make jokes about it or try to make light of it.**				
**11**	**When dealing with a problem, I make compromises.**				
**12**	**When dealing with a problem, I take steps to take better care of myself and my family for the future.**				
**13**	**When dealing with a problem, I work on making things better for the future by changing my habits, such as diet, exercise, budgeting, or staying in closer touch with people I care about.**				
